# Calcium imaging analysis – how far have we come?

**DOI:** 10.12688/f1000research.51755.1

**Published:** 2021-03-30

**Authors:** Miranda Robbins, Charles N. Christensen, Clemens F. Kaminski, Marta Zlatic

**Affiliations:** 1 MRC Laboratory of Molecular Biology, Cambridge, UK; 2 Department of Chemical Engineering and Biotechnology, University of Cambridge, Cambridge, UK

**Keywords:** Calcium Imaging, Denoising, Motion Correction, Classification, Quantification, Machine Learning, Neural Networks

## Abstract

Techniques for calcium imaging were first achieved in the mid-1970s, whilst tools to analyse these markers of cellular activity are still being developed and improved. For image analysis, custom tools were developed within labs and until relatively recently, software packages were not widely available between researchers. We will discuss some of the most popular, alongside our preferred, methods for calcium imaging analysis that are now widely available and describe why these protocols are so effective. We will also describe some of the newest innovations in the field that are likely to benefit researchers, particularly as calcium imaging is often an inherently low signal-to-noise method. Although calcium imaging analysis has seen recent advances, particularly following the rise of machine learning, we will end by highlighting the outstanding requirements and questions that hinder further progress, and pose the question of how far we have come in the past sixty years and what can be expected for future development in the field.

## Introduction

The ability to image calcium ion (Ca ^2+^) dynamics in cells has long been of interest, particularly in the neurosciences, where it can be used as a marker for neuronal excitability. The origins of calcium imaging began in the mid-1970s ( Blinks *et al.*, 1976; Moisescu *et al.*, 1975), however the most Ca ^2+^specific BAPTA-based dye was developed in 1980 by Roger Tsien, and its derivatives are still used today ( Tsien, 1980). In the past forty years, the methods available for measuring Ca ^2+^ fluxes in cells have expanded to include ratiometric, fluorescence lifetime, or fluorescence intensity, based reporters, and genetically-encoded options ( Miyawaki *et al.*, 1997; Ohkura *et al.*, 2005) alongside dyes. The use of microscopy modalities has also advanced to include light-sheet microscopy ( Huisken *et al.*, 2004) for long-term imaging, and two-photon microscopy ( Denk *et al.*, 1990) for deep tissue and cell specific uncaging techniques.

**Figure 1.**
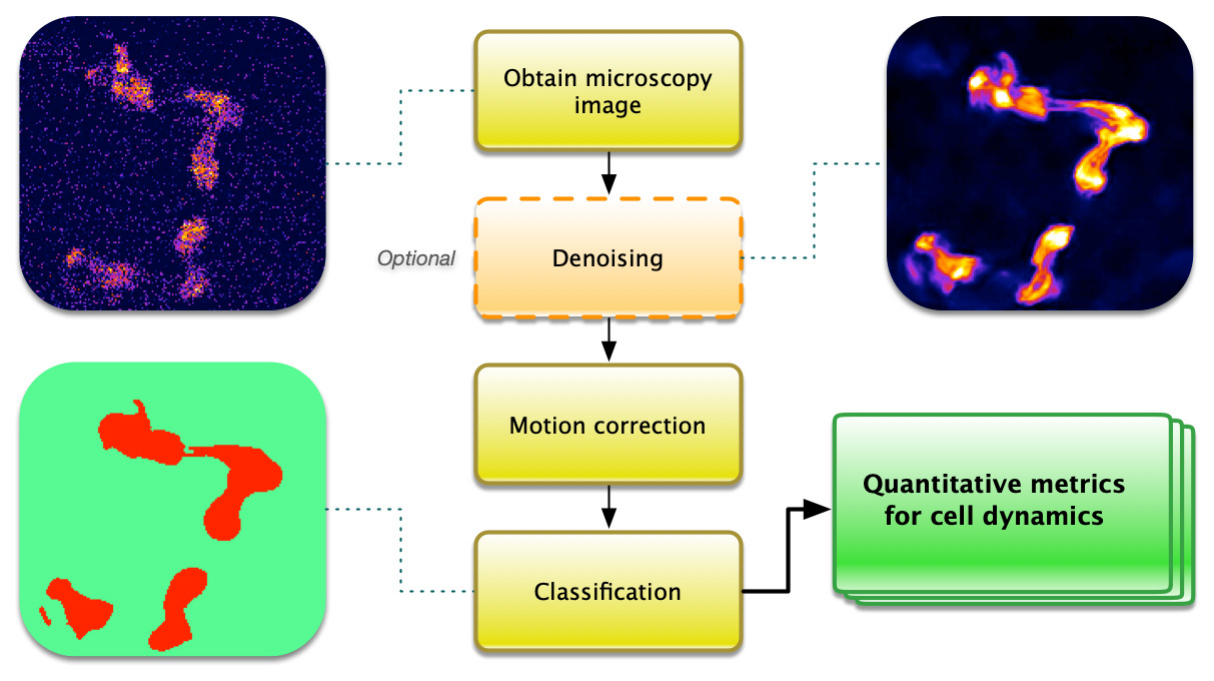
The steps of a common pipeline for calcium imaging analysis.

Calcium imaging is an inherently noisy method due to the high spatiotemporal information desired from a sample often showing low signal-to-noise alongside drift or cell movement, particularly for living organisms. In recent years, a number of software packages have been written for individual aspects of the commonly used pipeline in calcium imaging analysis ( Figure 1). This processing pipeline includes image denoising, motion correction, classification for cell identification, and quantification of calcium signals.

## Denoising

Although denoising is not a required step in the pipeline, effective denoising can improve the subsequent steps by artificially enhancing signal-to-noise. Traditionally, image denoising has been based on local averaging approaches, such as the application of a Gaussian smoothing filter ( Buades *et al*., 2005; Lindenbaum *et al.,* 1994). Other local filter methods include least mean squares filter ( Haykin & Widrow, 2003), anisotropic filters ( Perona & Malik, 1990) and in the frequency domain, Wiener filters ( Wiener, 1950) and wavelet thresholding methods ( Donoho, 1995).

Local methods are computationally light but have clear limitations. Firstly, the averaging often involved in local methods introduces blur, rendering features to be less defined. Secondly, they do not perform well for high noise levels, since the correlations between neighbouring pixels deteriorate ( Shao *et al.,* 2014).

Non-local filters solve some of these problems by using self-similarity of natural images beyond neighbouring pixels ( Shao *et al.,* 2014). The first method to propose this is the non-local means method ( Buades *et al.,* 2005), in which patches are restored by weighted averaging of all other patches in an image. Since then, there have been a number of improvements such as invariance to patches that are rotated or mirrored with respect to each other ( Grewenig *et al.,* 2011), improved computational efficiency, and automated parameter tuning and extension to 3D image stacks ( Coupé *et al.,* 2008). Although non-local filters are better at high noise levels, they will typically lead to artefacts like over-smoothing ( Shao *et al.,* 2014). A modern, well-balanced and state-of-the-art non-local method is ND-SAFIR, which is specifically geared towards application in fluorescence microscopy imaging ( Boulanger *et al.*, 2010). ND-SAFIR is a powerful method for removing Poisson-Gaussian noise, which is based on non-local means denoising ( Buades *et al.,* 2011) to first use a variance stabilisation step, followed by spatial and temporal patch-based weighted averages of intensity values. The method is widely applicable between experimental samples and can be used directly for 2D+t and 3D+t datasets.

In recent years, deep learning methods have become state-of-the-art for denoising. Methods such as DnCNN ( Zhang *et al.,* 2017), FFDNet ( Zhang *et al.,* 2018) and CARE ( Weigert *et al.,* 2018) rely on convolutional neural networks that are trained in a supervised learning approach. However, this requires ground truths to be available for model training, which may be difficult to obtain in practice. A different approach was developed in noise2noise ( Lehtinen *et al.,* 2018), where instead of learning the mapping from noisy images to clean targets, the model is trained with other noisy images as targets. The images must be corresponding pairs displaying the same objects but with independent noise. Assuming the noise sources underlying the images have zero-mean distributions, the weights of the network will then converge during training to the same values as a network trained with clean targets because the noise that manifests in the weights cancels out. A more recent method, noise2void ( Krull *et al.,* 2019), aims to resolve this issue of needing ground truths, by using self-supervised learning. Here, the network is optimised to predict the value of each pixel from the values of neighbouring pixels in an image, thus requiring no separate ground truths.

## Motion correction

Motion correction can be split into two main categories, which may be selected depending on the experimental model. Many samples will face drift during imaging or shift when imaging the same field of view over multiple days. which can be well rectified using standard registration methods ( Thévenaz *et al.*, 1998).

More complex motion such as organism movement can be harder to correct as it is often non-uniform, over a large area, and causes movement in-and-out of the focal plane. These require non-rigid registration methods or motion tracking. A commonly used example available in Python and MATLAB is Non-Rigid Motion Correction, NoRMcorre ( Pnevmatikakis & Giovannucci, 2017), which uses patch-based field of view registration whereby separate images are then merged by smooth interpolation. The popularity of NoRMcorre may in part be due to its general applicability.

Two correction methods have been produced for 2-photon *in vivo* imaging in awake rodents, one based on the Lucas–Kanade (gradient descent) image registration algorithm using MathWorks® MATLAB platform ( Greenberg & Kerr, 2009), the other using a Hidden Markov Model ( Dombeck *et al.*, 2007). Although effective, these methods have not been packaged for easy implementation and are reliant in cells remaining in the x- and y- dimensions as it cannot track following movement between z-axes. In cases with z-axis movement, tracking-based methods may be more reliable, and specialist options exist using control theory and machine learning approaches for post-processing ( Nguyen *et al.*, 2017), or applied to a motorised stage ( Cong *et al.*, 2017; Kim *et al.*, 2017).

Tracking methods specifically designed to be more basic to implement and widely available include plug-ins for image processing packages ( Abramoff *et al.,* 2004) such as Trackmate ( Tinevez *et al.*, 2017), or Time Series Analyzer (Balaji, UCLA).

## Classification

Classification can be achieved through pixel- or object-based segmentation. Pixel-based methods map each pixel to a class according to the spectral similarities. Popular pixel-based methods for calcium image analysis include Maximum Likelihood Classification (MLC) or Otsu thresholding to separate ‘light’ and ‘dark’ clustered pixels ( Otsu, 1979) as used as part of the SIMA Python package ROI pipeline ( Kaifosh *et al.*, 2014).

Object-based segmentation is a two-step process using both spectral and spatial/contextual information to group pixels into objects which are then classified. CaImAn is an open-source classification method based on convolutional neural networks ( Giovannucci *et al.*, 2019). It was packaged into EZcalcium in an effort to improve usability by providing a GUI in MathWorks® MATLAB ( Cantu *et al.*, 2020). However, using limited CaImAn function in EZcalcium does not easily allow for segmentation of more complex structures or large organelles or clusters of cells and is better for somas or smaller, less complex areas. Cellpose is another generalist, deep learning-based segmentation method that uses entirely open source packages in Python with a GUI to aid implementation. There is also a web-based option for testing Cellpose, which makes it very easy to use ( Stringer *et al.,* 2020), though it too can be limited at detecting more complex cell shapes such as dendrites and axons.

DenoiSeg is an extension of Noise2Void that offers an end-to-end neural network, which is jointly optimised to denoise and segment images. The denoising capability is learnt by the self-supervised learning principle that noise2void introduced ( Krull *et al.,* 2019). By combining this with a supervised learning approach using a few annotated ground truths of segmentation maps, the final segmentation performance ends up performing better than without co-learning, i.e. having two separate networks perform the respective tasks ( Buchholz *et al.,* 2020).

Cell classification methods have been discussed with the conclusion that ‘learning-based methods score among the best-performing methods, but well-optimized traditional methods can even surpass these approaches in a fraction of the time’ ( Vicar *et al.*, 2019).

## Quantification

The aim of each step is for signal extraction to allow a quantitative output from the images of calcium signals. The most commonly used measure is the relative fluorescence variation (ΔF/F0) for classified cells. Packages will therefore either provide this data for further analysis, or provide a direct plot. Background subtraction may need to be considered as not all packages will take this into account.

Another feature commonly needed by researchers is timing of neuronal action potentials or ‘spike detection’. A wide range of algorithms can be used as discussed in the results to the *Spikefinder* challenge ( Berens *et al.*, 2018) as there are multiple methods of varying complexity that can be used. EZcalcium directly shows the raw fluorescence, inferred activity and deconvolved neural ‘spiking’, whereby the data can then be exported into file formats for proprietary (.mat, .xlsx) or open (.csv) software programmes for further analysis ( Cantu *et al.*, 2020; Giovannucci *et al.*, 2019).

## Conclusion

A great number of analysis advancements have been made since calcium imaging was first developed. Popular packages for various steps of the pipeline ( Figure 1) include CaImAn, SIMA, Suite2P, and EZcalcium ( Cantu *et al*., 2020; Giovannucci *et al*., 2019; Kaifosh *et al.*, 2014; Pachitariu *et al.*, 2016). Although these packages are great starting tools for the community, many require programming knowledge in Python or commercial packages such as MathWorks® MATLAB. Many of the available options are only semi-automated and the limited automated options available are often designed for a very limited experimental context and are not actively supported when problems are experienced, e.g. other than for cells of a specific size and shape imaged *in vitro*. Suite2P and EZcalcium both attempt to offer an automated pipeline from raw images to spike extraction ( Cantu *et al.*, 2020; Pachitariu *et al.*, 2016). EZcalcium is one of the most intuitive options, which has improved the usability of CaImAn, NoRMCorre, but again seems best suited to analyse cell bodies.

It therefore seems that perhaps some of the biggest advances could be made by designing packages for detecting neuritic structures or organelles and improving the spatial resolution of the analysis to be intracellular, such as has been used for calcium sparks ( Berens *et al.*, 2018). On the other end of the scale, pipelines for functional imaging in organisms such as zebrafish, *C. elegans* and *Drosophila*, where motion correction is often required and improved analysis for connectomics purposes are much needed.

As the application of machine learning in calcium imaging analysis matures, a higher level of automation and throughput for analysis tasks can be expected to follow. This will be enabled by more generalised and robust machine learning models. The barrier to training and deploying these methods will also reduce as more research is made into few-shot learning (using small training datasets) in addition to training approaches such as self-supervised and unsupervised learning.

## Data availability

No data are associated with this article.
