# Calcium imaging analysis – how far have we come?

**DOI:** 10.12688/f1000research.51755.2

**Published:** 2021-08-26

**Authors:** Miranda Robbins, Charles N. Christensen, Clemens F. Kaminski, Marta Zlatic

**Affiliations:** 1 MRC Laboratory of Molecular Biology, Cambridge, UK; 2 Department of Chemical Engineering and Biotechnology, University of Cambridge, Cambridge, UK

**Keywords:** Calcium Imaging, Denoising, Motion Correction, Classification, Quantification, Machine Learning, Neural Networks

## Abstract

Techniques for calcium imaging were first demonstrated in the mid-1970s, whilst tools to analyse these markers of cellular activity are still being developed and improved today. For image analysis, custom tools were developed within labs and until relatively recently, software packages were not widely available between researchers. We will discuss some of the most popular methods for calcium imaging analysis that are now widely available and describe why these protocols are so effective. We will also describe some of the newest innovations in the field that are likely to benefit researchers, particularly as calcium imaging is often an inherently low signal-to-noise method. Although calcium imaging analysis has seen recent advances, particularly following the rise of machine learning, we will end by highlighting the outstanding requirements and questions that hinder further progress and pose the question of how far we have come in the past sixty years and what can be expected for future development in the field.

## Introduction

The ability to image calcium ion (Ca ^2+^) dynamics in cells has long been of interest, particularly in the neurosciences, where it can be used as a marker for neuronal excitability. The origins of calcium imaging began in the mid-1970s ( Blinks *et al.*, 1976; Moisescu *et al.*, 1975), however the most Ca ^2+^ specific BAPTA (1,2-bis(o-aminophenoxy)ethane-N,N,N′,N′-tetraacetic acid)-based dye was developed in 1980 by Roger Tsien, and its derivatives are still used today ( Tsien, 1980). In the past forty years, the methods available for measuring Ca ^2+^ fluxes in cells have expanded to include ratiometric, fluorescence lifetime, or fluorescence intensity-based dyes, and genetically-encoded calcium indicators (GECIs) ( Miyawaki *et al.*, 1997; Ohkura *et al.*, 2005). The use of microscopy modalities has also advanced to include light-sheet microscopy (LSM; Huisken *et al.*, 2004) for long-term imaging, and 2-photon microscopy (2PM; Denk *et al.*, 1990) for deep tissue and cell specific uncaging techniques. The combination of Ca ^2+^ indicator and imaging modality used will reflect the properties of the sample and the scientific question, as well as the methodologies available to the researcher. For example, *in vitro* imaging, or *in vivo* invertebrate imaging, may use exogenous or GECIs, imaged using LSM, epifluorescence, 2PM or other fluorescence microscopes depending on the temporal and spatial resolution, timescale of imaging, and thickness of the sample being taken into consideration. Other specialist options for *in vivo* imaging of GECIs are available for imaging in awake and behaving animals including miniaturized forms of 1- or 2- photon endoscopic fluorescence microscopes (miniscopes) for single-cell *in vivo* recordings ( Cai *et al.*, 2016; Chen *et al.*, 2013; Silva, 2017).

**Figure 1.**
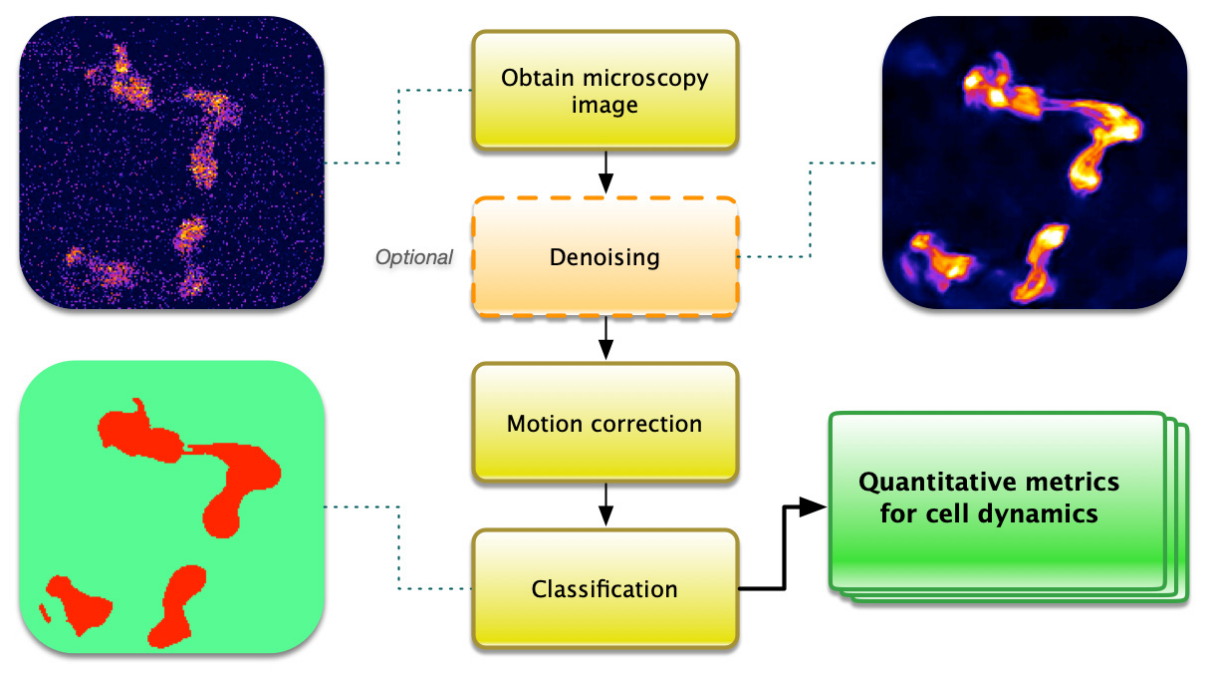
The steps of a common pipeline for calcium imaging analysis can be subdivided into three areas before quantitative analysis is performed. Denoising is an optional step that can help to improve signal-to-noise and enhance features. Motion correction may be necessary in cases of drift or movement. Classification can select regions of interest for which quantitative analysis is performed.

Calcium imaging is an inherently noisy method due to the high spatiotemporal information desired from a sample often showing low signal-to-noise alongside drift or cell movement, particularly for living organisms. In recent years, a number of software packages have been written for individual aspects of the commonly used pipeline in calcium imaging analysis ( Figure 1). This processing pipeline includes image denoising, motion correction, classification for cell identification, and quantification of calcium signals. As calcium imaging is used across a broad range of samples, from sub-cellular, cellular, networks, bulk tissue dynamics to whole organisms and behaving animals, aspects of this pipeline can vary substantially with no ‘one size fits all’ approach.

## Denoising

Live-cell imaging generally requires short exposure times and low excitation power to limit the effects of photo-toxicity and photo-bleaching. This leads to image degradation in the form of noise. In fluorescence microscopy the two prevalent noise sources are Poisson noise and Gaussian noise. Poisson noise is caused by the stochastic and discrete nature of photon emission and tends to be dominant at low light levels, whereas Gaussian noise describes the intrinsic thermal and electronic fluctuation in the image sensor ( Luisier *et al.*, 2011)

Although denoising is not a required step in the pipeline, effective denoising can improve the subsequent steps by artificially enhancing signal-to-noise. Traditionally, image denoising has been based on local averaging approaches, such as the application of a Gaussian smoothing filter ( Buades *et al*., 2005; Lindenbaum *et al.,* 1994). Alternative methods include a local filter method such as anisotropic filters ( Broser *et al.*, 2004; Kitamura & Häusser, 2011) ( Perona & Malik, 1990), or in the frequency domain, Wiener filters ( Wiener, 1950) and wavelet thresholding methods ( Donoho, 1995)( Besbeas *et al.*, 2004; Wegner *et al.*, 2006).

Local methods are computationally light but have clear limitations. First, the averaging often involved in local methods introduces blur, causing features to appear less well defined. Second, they do not perform well for high noise levels, since the correlations between neighbouring pixels deteriorate ( Shao *et al.,* 2014). In the context of calcium imaging, local methods have been shown to perform well ( Malik *et al.*, 2011).

Non-local filters solve some of the problems by using self-similarity of natural images beyond neighbouring pixels, thus exploiting global information ( Shao *et al.,* 2014). The first method to propose this is the non-local means method ( Buades *et al.,* 2005), in which subregions of an image referred to as patches are restored by weighted averaging of all other patches in an image. Since then, there have been a number of improvements such as invariance to patches that are rotated or mirrored with respect to each other ( Grewenig *et al.,* 2011), improved computational efficiency, and automated parameter tuning and extension to 3D image stacks ( Coupé *et al.,* 2008). Although non-local filters are better at high noise levels, they will typically lead to artefacts like over-smoothing ( Shao *et al.,* 2014). A modern, well-balanced and state-of-the-art non-local method is ND-SAFIR, which is specifically geared towards application in fluorescence microscopy imaging ( Boulanger *et al.*, 2010). ND-SAFIR is a powerful method for removing Poisson-Gaussian noise. It is based on non-local means denoising using a variance stabilisation step, followed by calculating the spatial and temporal patch-based weighted averages for intensity values. The method is widely applicable between experimental samples and can be used directly for 2D+t and 3D+t datasets ( Buades *et al.,* 2011).

In recent years, deep learning methods have become state-of-the-art for denoising. Methods such as DnCNN ( Zhang *et al.,* 2017), FFDNet ( Zhang *et al.,* 2018) and CARE ( Weigert *et al.,* 2018) rely on convolutional neural networks that are trained in a supervised learning approach. However, this requires ground truths to be available for model training, which may be difficult to obtain in practice. A different approach was developed in noise2noise ( Lehtinen *et al.,* 2018), where instead of learning the mapping from noisy images to clean targets, the model is trained with other noisy images as targets. The images must be corresponding pairs displaying the same objects but with independent noise. Assuming the noise sources underlying the images have zero-mean distributions, the weights of the network will then converge during training to the same values as a network trained with clean targets because the noise that manifests in the weights cancels out. A more recent method, noise2void ( Krull *et al.,* 2019), aims to resolve this issue of needing ground truths, by using self-supervised learning. Here, the network is optimised to predict the value of each pixel from the values of neighbouring pixels in an image, thus requiring no separate ground truths. In another recent method, DeepInterpolation ( Lecoq *et al.*, 2020), the need for ground truth training data is avoided by treating the denoising task as a nonlinear interpolation problem. This assumes that the data have a sequential component, such that spatiotemporally overlapping features can be exploited. DeepCAD is a new deep self-supervised denoising method that reduces detection noise and thereby improve the signal-to-noise more than tenfold, which it claims can improve the accuracy of neuron extraction and spike inference ( Li *et al.*, 2021).

## Motion correction

Motion correction is often required to ensure consistent image processing across a time stack. We distinguish between two types of motion: (a) drift occurring in the imaging system itself caused by thermal gradients in the microscope, vibrations and mechanical instability ( Kreft *et al.*, 2005); (b) subject motion such as fluctuations in the immersion media or the movement of organisms ( Jenkinson *et al.*, 2002). Drift will typically play a significant role when imaging the same field of view over multiple days, which can be rectified by using standard registration methods ( Dubbs *et al.*, 2016)( Thévenaz *et al.*, 1998).

More complex motion such as organism movement can be harder to correct as it is often non-uniform, over a large area, and causes movement in-and-out of the focal plane. These require non-rigid registration methods or motion tracking. A commonly used example available in Python and MATLAB is Non-Rigid Motion Correction, NoRMcorre ( Pnevmatikakis & Giovannucci, 2017), which uses patch-based field of view registration whereby separate images are then merged by smooth interpolation. The popularity of NoRMcorre may in part be due to its general applicability.

Two correction methods have been produced for *in vivo* imaging in awake rodents, one based on the rigid-transform-based Lucas–Kanade (gradient descent) ( Lucas & Kanade, 1981) image registration algorithm using MathWorks® MATLAB platform ( Greenberg & Kerr, 2009), the other using a Hidden Markov Model ( Dombeck *et al.*, 2007). Although effective, these methods have not been packaged for easy implementation and are reliant on cells remaining in the x- and y- dimensions as it cannot track following movement between z-axes. In cases with z-axis movement, tracking-based methods may be more reliable, and specialist options exist using control theory and machine learning approaches for post-processing ( Nguyen *et al.*, 2017), or applied to a motorised stage ( Cong *et al.*, 2017; Kim *et al.*, 2017). A MathWorks® MATLAB toolbox, miniscope 1-photon imaging pipeline (MIN1PIPE), has been developed to include denoising, motion correction and signal extraction ( Lu *et al.*, 2018). MIN1PIPE motion correction includes several steps including the Lucas-Kanade and Kanade-Lucas-Tomasi ( Lucas & Kanade, 1981; Shi & Tomasi, 1994) trackers, and Log-Demons registration ( Vercauteren *et al.*, 2009), and outperforms the Lucas-Kanade, Kanade-Lucas-Tomasi, and NoRMcorre for using sample 2-photon videos ( Lu *et al.*, 2018).

Tracking methods specifically designed to be more basic to implement and widely available include plug-ins for image processing packages ( Abramoff *et al.,* 2004) such as Trackmate ( Tinevez *et al.*, 2017), or Time Series Analyzer ( Balaji, 2014).

## Classification

Classification is required to ensure that the quantification can be performed over specific regions of interest, such as for subcellular area, specific cells, or tissue regions. Classification can be achieved through pixel- or object-based segmentation. Pixel-based methods map each pixel to a class according to the spectral similarities. Popular pixel-based methods for calcium image analysis include Maximum Likelihood Classification (MLC) ( Malik *et al.*, 2011) or Otsu thresholding to separate ‘light’ and ‘dark’ clustered pixels ( Otsu, 1979) as used as part of the SIMA Python package ROI pipeline ( Kaifosh *et al.*, 2014).

Object-based segmentation is a two-step process using both spectral and spatial/contextual information to group pixels into objects which are then classified. CaImAn is an open-source package with modules for classification, motion correction, source extraction, and spike deconvolution. The classification method is based on convolutional neural networks ( Giovannucci *et al.*, 2019). It was packaged into EZcalcium in an effort to improve usability by providing a GUI in MathWorks® MATLAB ( Cantu *et al.*, 2020). However, using limited CaImAn function in EZcalcium does not easily allow for segmentation of more complex structures or large organelles or clusters of cells and is better for somas or smaller, less complex areas. Cellpose is another generalist, deep learning-based segmentation method that uses entirely open source packages in Python with a GUI to aid implementation. There is also a web-based option for testing Cellpose, which makes it very easy to use ( Stringer *et al.*, 2020), though it too can be limited at detecting more complex cell shapes such as dendrites and axons.

DenoiSeg is an extension of Noise2Void that offers an end-to-end neural network, which is jointly optimised to denoise and segment images. The denoising capability is learnt by the self-supervised learning principle that noise2void introduced ( Krull *et al.,* 2019). By combining this with a supervised learning approach using a few annotated ground truths of segmentation maps, the final segmentation performance ends up performing better than without co-learning, i.e. having two separate networks perform the respective tasks ( Buchholz *et al.,* 2020).

Cell classification methods have been discussed with the conclusion that ‘learning-based methods score among the best-performing methods, but well-optimized traditional methods can even surpass these approaches in a fraction of the time’ ( Vicar *et al.*, 2019).

## Quantification

The aim of each step is for signal extraction to allow a quantitative output from the images of calcium signals. The most commonly used measure is the relative fluorescence variation (ΔF/F0) for classified cells. Packages will therefore either provide this data of the baseline fluorescence (F0) and deviations from baseline (ΔF), for further analysis, or provide a direct plot. Background subtraction may need to be considered as not all packages will take this into account. Multiple methods can be used, including subtracting the intensity values from a region of the image that does not contain Ca ^2+^ indicator from the intensity values in regions of interest. However care should be taken using background subtraction with ratiometric indicators ( Shkryl, 2020). F0 baseline values can be calculated by averaging the values before the onset of stimulation in the same region ( Galizia *et al.*, 1999), or by low-pass filtering the signal ( Balkenius *et al.*, 2009) (For review ( Balkenius *et al.*, 2015).

Signal extraction from single cells can be particularly difficult for *in vivo* recordings due to large background fluxes and high spatial overlaps of cells outside of the focus plane which is further increased in 1-photon compared to 2-photon imaging. Semi-automated ROI analysis ( Barbera *et al.*, 2016; Klaus *et al.*, 2017; Pinto & Dan, 2015), principal component analysis independent components analysis (PCA/ICA) ( Mukamel *et al.*, 2009), clustering based approaches (like Suite2P; ( Pachitariu *et al.*, 2017), and constrained nonnegative matrix factorization (CNMF) ( Pnevmatikakis *et al.*, 2016) approaches are techniques that have been explored with different strengths for detecting background and spatial overlap. An ‘extended’ CNMF method (CNMF-E) has been developed with an adjusted spatiotemporal background model that outperformed PCA/ICA for the simulated and experimental datasets that were tested ( Zhou *et al.*, 2018). For a package method, the toolbox MIN1PIPE combines a CNMF ( Pnevmatikakis *et al.*, 2016) with additional steps to remove false positives ( Lu *et al.*, 2018). CaImAn also builds upon the CNMF algorithm ( Pnevmatikakis *et al.*, 2016) to allow it to be fully automated, and CNMF-E for 1-photon endoscopic data ( Zhou *et al.*, 2018).

Another feature commonly needed by researchers is timing of neuronal action potentials (APs) or ‘spike detection’ through deconvolution of the extracted signal. A wide range of algorithms can be used as discussed in the results to the *Spikefinder* challenge ( Berens *et al.*, 2018) as there are multiple methods of varying complexity that can be used. EZcalcium directly shows the raw fluorescence, inferred activity and deconvolved neural ‘spiking’, whereby the data can then be exported into file formats for proprietary (.mat, .xlsx) or open (.csv) software programmes for further analysis ( Cantu *et al.*, 2020; Giovannucci *et al.*, 2019). The ability to accurately detect spikes requires knowledge of ground truth, usually from electrophysiological recordings. Calcium imaging can be susceptible to variation between neuron type, calcium indicator and its concentration used, the optical resolution, the sampling rate and the noise level. Therefore, it is fundamental to understand how specific indicators react under the given imaging conditions, which cannot be readily generalized across protocols. To try and improve the accuracy of spike detection, a toolkit using a supervised algorithm of spike inference has been developed using a ‘ground truth database’ from a large number of sets of calcium imaging with corresponding electrophysiological measurements ( Rupprecht *et al.*, 2021).

## Conclusion

A great number of analysis advancements have been made since calcium imaging was first developed. Popular packages for various steps of the pipeline ( Figure 1) include CaImAn, SIMA, Suite2P, and EZcalcium ( Cantu *et al*., 2020; Giovannucci *et al*., 2019; Kaifosh *et al.*, 2014; Pachitariu *et al.*, 2017). Although these packages are great starting tools for the community, many require programming knowledge in Python or commercial packages such as MathWorks® MATLAB. Many of the available options are only semi-automated and the limited automated options available are often designed for a very limited experimental context and are not actively supported when problems are experienced, e.g. other than for cells of a specific size and shape imaged *in vitro*. EZcalcium is one of the most intuitive options, which has improved the usability of CaImAn, NoRMCorre, but again seems best suited to analyse cell bodies. Suite2P and EZcalcium both attempt to offer an automated pipeline from raw images to spike extraction with no prior programming knowledge required by the user ( Cantu *et al.*, 2020; Pachitariu *et al.*, 2017). As both packages are suited to similar experimental data, the choice may be based upon personal preference.

It therefore seems that perhaps some of the biggest advances could be made by designing packages for detecting neuritic structures or organelles and improving the spatial resolution of the analysis to be intracellular, such as has been used for calcium sparks ( Berens *et al.*, 2018). Longitudinal tracking of specific cells across imaging sessions also remains a challenge so that individual cells can be identified between multiple imaging sessions. A MathWorks® MATLAB toolkit has been made with reported error rates of < 5 % ( Sheintuch *et al.*, 2017); an alternative approach is also available using CaImAn ( Giovannucci *et al.*, 2019) though direct comparisons between these methods is difficult without knowing ground truths. Calcium imaging for population activity has also been highlighted as an area that requires further research, particularly when imaging over larger fields of view. Using models specific for neuron types imaged may improve detection of APs, which are commonly under-represented in population activity measurements ( Huang *et al.*, 2021). Recent toolboxes with large datasets containing ground-truths may reduce false negatives during analysis ( Rupprecht *et al.*, 2021). On the other end of the scale, pipelines for functional imaging in organisms such as zebrafish, *C. elegans* and *Drosophila*, where motion correction is often required and improved analysis for connectomics purposes are much needed.

As the application of machine learning in calcium imaging analysis matures, a higher level of automation and throughput for analysis tasks can be expected to follow. This will be enabled by more generalised and robust machine learning models. The barrier to training and deploying these methods will also reduce as more research is made into few-shot learning (using small training datasets) in addition to training approaches such as self-supervised and unsupervised learning.

## Data availability

No data are associated with this article.
