# Supplementary material for: Calcium imaging analysis – how far have we come?
Source: F1000Res. 2021 Aug 26;10:258. Originally published 2021 Mar 30. [Version 2] doi: 10.12688/f1000research.51755.2 (PMC8406438; doi:10.12688/f1000research.51755.2)
Supplement: Peer review history

# Peer review: Reviewer response for version 2

**DOI:** 10.5256/f1000research.76662.r92941

**Published:** 2021-09-16

**Authors:** Anubhuti Goel

**Affiliations:** 1 Department of Psychology, University of California, Riverside (UCR), Riverside, CA, USA

The authors have addressed my concerns.

Is the review written in accessible language?

Yes

Are all factual statements correct and adequately supported by citations?

Partly

Are the conclusions drawn appropriate in the context of the current research literature?

Yes

Is the topic of the review discussed comprehensively in the context of the current literature?

Yes

Reviewer Expertise:

Systems neuroscience, Calcium imaging, mouse behavior, electrophysiology.

I confirm that I have read this submission and believe that I have an appropriate level of expertise to confirm that it is of an acceptable scientific standard.

# Peer review: Reviewer response for version 2

**DOI:** 10.5256/f1000research.76662.r92939

**Published:** 2021-09-02

**Authors:** Christopher Rowlands, Isabell Whiteley

**Affiliations:** 1 Department of Bioengineering, Imperial College London, London, UK

We would like to thank the authors for responding to all of our points stated in the first review. While there are still some parts where improvements could still be made, these issues are comparatively minor, and thus we feel this paper now has a more complete review of the steps required to detect calcium signals in neurons.

 Overall, this review provides important information for researchers using calcium imaging, and can be used as a resource for finding analysis tools that fit their needs. The new definitions provided give further clarity to the concepts and tools discussed in the paper, and the up to date citations will direct the reader to papers that have applied these tools to their research.  After all these changes and additions to the manuscript, we have no further corrections and feel that the paper is ready for indexing.

Is the review written in accessible language?

Partly

Are all factual statements correct and adequately supported by citations?

Yes

Are the conclusions drawn appropriate in the context of the current research literature?

Yes

Is the topic of the review discussed comprehensively in the context of the current literature?

Yes

Reviewer Expertise:

Microscopy, instrument development, automation and data analysis, neurophotonics, biophotonics

We confirm that we have read this submission and believe that we have an appropriate level of expertise to confirm that it is of an acceptable scientific standard.

# Peer review: Reviewer response for version 2

**DOI:** 10.5256/f1000research.76662.r92940

**Published:** 2021-08-27

**Authors:** William Zeiger

**Affiliations:** 1 Department of Neurology, University of California, Los Angeles (UCLA), Los Angeles, CA, USA

The authors have adequately addressed all suggested critiques. I have no further comments.

Is the review written in accessible language?

Yes

Are all factual statements correct and adequately supported by citations?

Yes

Are the conclusions drawn appropriate in the context of the current research literature?

Yes

Is the topic of the review discussed comprehensively in the context of the current literature?

Partly

Reviewer Expertise:

Stroke, neural circuits, two-photon calcium imaging

I confirm that I have read this submission and believe that I have an appropriate level of expertise to confirm that it is of an acceptable scientific standard.

# Peer review: Reviewer response for version 1

**DOI:** 10.5256/f1000research.54953.r84795

**Published:** 2021-06-07

**Authors:** Anubhuti Goel

**Affiliations:** 1 Department of Psychology, University of California, Riverside (UCR), Riverside, CA, USA

The review by Miranda et al provides an overview of the calcium imaging pipeline and the open source resources available to the scientific community. Overall the review is a helpful resource to anyone aiming to analyze their calcium imaging data. However a clearer understanding of which resource is better suited to which model system or type of analysis will be a helpful addition (maybe some examples will be particularly insightful). The authors mention Suite2P and EZCalcium as the top analysis tools available however it is not clear which one the reader should pick. Furthermore both Suite2P and EZCalcium come with a user interface which means other than downloading either python or MATLAB no programming knowledge is needed. This fact is not clear in the review.

 One reference is mentioned as "Balaji, UCLA". The authors  should specify if indeed that is a personal communication or perhaps an unpublished tool.

Is the review written in accessible language?

Yes

Are all factual statements correct and adequately supported by citations?

Partly

Are the conclusions drawn appropriate in the context of the current research literature?

Yes

Is the topic of the review discussed comprehensively in the context of the current literature?

Yes

Reviewer Expertise:

Systems neuroscience, Calcium imaging, mouse behavior, electrophysiology.

I confirm that I have read this submission and believe that I have an appropriate level of expertise to confirm that it is of an acceptable scientific standard, however I have significant reservations, as outlined above.

# Response

**Published:** 2021-08-16

**Authors:** Miranda Robbins

**Affiliations:** MRC Laboratory of Molecular Biology, UK

The review by Miranda et al provides an overview of the calcium imaging pipeline and the open source resources available to the scientific community. Overall the review is a helpful resource to anyone aiming to analyze their calcium imaging data.

*We thank the reviewer for their helpful suggestions for our article.*

 However a clearer understanding of which resource is better suited to which model system or type of analysis will be a helpful addition (maybe some examples will be particularly insightful).

*We have included in the introduction information about the methods that may better suit in vitro, versus in vivo imaging methods:*

**“The combination of Ca ^2+^ indicator and imaging modality used will reflect the properties of the sample and the scientific question, as well as the methodologies available to the researcher. For example, *in vitro* imaging, or *in vivo* invertebrate imaging, may use exogenous or GECIs, imaged using LSM, epifluorescence, 2PM or other fluorescence microscopes depending on the temporal-resolution, timescale of imaging, and thickness of the sample being taken into consideration. Other specialist options for *in* *vivo *imaging of GECIs are available for imaging in awake and behaving animals including miniaturized forms of 1- or 2- photon endoscopic fluorescence microscopes (miniscopes) for single-cell in *vivo* recordings** **(Cai et al., 2016; Chen et al., 2013; Silva, 2017)** **.”**

*We have added more information for 1 photon in vivo imaging paragraph in ‘motion correction’ section.*

*We have added more information to the in vivo quantification section:*

**“Signal extraction from single cells can be particularly difficult for in vivo recordings due to large background fluxes and high spatial overlaps of cells outside of the focus plane. Semi-automated ROI analysis (Pinto and Dan, 2015; Barbera et al., 2016; Klaus et al., 2017), principal component analysis independent components analysis (PCA/ICA) (Mukamel, Nimmerjahn and Schnitzer, 2009), and constrained nonnegative matrix factorization (CNMF) (Pnevmatikakis et al., 2016; Zhou et al., 2018) approaches are techniques that have been explored with different strengths for detecting background and spatial overlap. An ‘extended’ CNMF method has been developed with an adjusted spatiotemporal background model that outperformed PCA/ICA for the simulated and experimental datasets that were tested (Zhou et al., 2018). For a package method, the toolbox MIN1PIPE combines a CNMF (Pnevmatikakis et al., 2016) with additional steps to remove false positives (Lu et al., 2018).”**

 The authors mention Suite2P and EZCalcium as the top analysis tools available however it is not clear which one the reader should pick. Furthermore both Suite2P and EZCalcium come with a user interface which means other than downloading either python or MATLAB no programming knowledge is needed. This fact is not clear in the review.

*We have added the following in the conclusion:*

**“Suite2P and EZcalcium both attempt to offer an automated pipeline from raw images to spike extraction with no prior programming knowledge required by the user ( Cantu et al., 2020; Pachitariu et al., 2016). As both packages are suited to similar experimental data, the choice may be based upon personal preference.”**

 One reference is mentioned as "Balaji, UCLA". The authors  should specify if indeed that is a personal communication or perhaps an unpublished tool.

*We understand that this may cause confusion; as this plug-in is not published and we have now added the ImageJ webpage as a reference.*

# Peer review: Reviewer response for version 1

**DOI:** 10.5256/f1000research.54953.r84794

**Published:** 2021-05-24

**Authors:** William Zeiger

**Affiliations:** 1 Department of Neurology, University of California, Los Angeles (UCLA), Los Angeles, CA, USA

In their review “Calcium imaging analysis – how far have we come?”, Robbins et al. present a concise overview of the basic steps involved in transforming raw microscopy images from calcium imaging experiments to quantifiable data. This is an extremely important topic with broad relevance to many fields. As such, the review should draw considerable interest. The review is well written. It is logically organized, with the analysis pipeline broken down into 4 distinct steps, each of which is discussed separately. The language is easy to follow for a broad audience of varying degrees of expertise. Many of the most popular software implementations are covered at least to some degree. Overall, I think this will be a useful and important review with significant impact.

 However, I do have some suggestions that I believe could strengthen the manuscript. My main concern is that the target audience for the review is not clear. Calcium imaging is broadly used in many areas of the life sciences, including both in vitro and in vivo preparations, across multiple species, multiple tissues, and widely varying spatial scales (sub-cellular, cellular, bulk tissue signals). Given that much of the review is devoted to denoising and motion correction (problems which are relatively minor in in vitro preparations with no organismal movement and relatively high signal-to-noise), and that quantification focuses on spike detection, the review feels most well suited to in vivo two-photon imaging applications in the brain. If this was the intention, it would benefit the review to make this more explicit and provide more discussion tailored to this technique, particularly in the quantification section. If this assumption is not correct and the review is meant to be targeted at a more general audience, I would suggest the introduction be expanded to include at least a brief discussion of the types of calcium indicators available (ratiometric, FRET, fluo, GCaMP, etc) and imaging techniques in use (widefield fluorescence, confocal, 2P, miniscopes, etc). This is important for a beginner/generalist audience as the indicator and imaging technique used will strongly influence the subsequent processing pipeline needs.

 In addition to this primary concern I have a few other suggestions that would improve the scope of the review:

- The quantification section should be significantly expanded. This step is the shortest in description, yet arguably can be fraught with the most pitfalls to which investigators fall prey.
- CaImAn is included under the “classification” section, but includes modules to do more than just classify, including motion correction and registration across imaging sessions
- Spike detection is mentioned, but it should be made explicit that spike detection is completely dependent on knowledge of ground truth about how specific indicators under specific imaging conditions relate and cannot be readily generalized across labs/indicators/preparations. See this recent preprint (Rupprecht et. al https://www.biorxiv.org/content/10.1101/2020.08.31.272450v2 ^1^)
- On a related point about spike detection, the authors may want to include information about the relative sensitivity (or in some cases lack thereof) of calcium indicators for spike detection (see Hunag et. al https://elifesciences.org/articles/51675 ^2^).
- It would be interesting for the authors to comment on 1P miniscope calcium imaging, as this technique is rapidly disseminating and poses specific challenges for imaging analysis. See Lu et. al ( https://www.cell.com/cell-reports/fulltext/S2211-1247(18)30826-X ^3^)
- Longitudinal tracking of cells across imaging sessions remains a challenge for the field – I am not aware of many algorithms that are widely available to tackle this problem. It could be included in the future directions.
- The Allen institute recently published a preprint on a new denoising technique which looks promising (See Lecoq et. al https://www.biorxiv.org/content/10.1101/2020.10.15.341602v2 ^4^)

Is the review written in accessible language?

Yes

Are all factual statements correct and adequately supported by citations?

Yes

Are the conclusions drawn appropriate in the context of the current research literature?

Yes

Is the topic of the review discussed comprehensively in the context of the current literature?

Partly

Reviewer Expertise:

Stroke, neural circuits, two-photon calcium imaging

I confirm that I have read this submission and believe that I have an appropriate level of expertise to confirm that it is of an acceptable scientific standard, however I have significant reservations, as outlined above.

## References

[1] : Database and deep learning toolbox for noise-optimized, generalized spike inference from calcium imaging. *bioRxiv*.2020; 10.1101/2020.08.31.27245010.1101/2020.08.31.272450PMC761161834341584

[2] : Relationship between simultaneously recorded spiking activity and fluorescence signal in GCaMP6 transgenic mice.*Elife*.2021;10: 10.7554/eLife.5167510.7554/eLife.5167533683198PMC8060029

[3] : MIN1PIPE: A Miniscope 1-Photon-Based Calcium Imaging Signal Extraction Pipeline. *Cell Reports*.2018;23(12) : 10.1016/j.celrep.2018.05.0623673-368410.1016/j.celrep.2018.05.062PMC608448429925007

[4] : Removing independent noise in systems neuroscience data using DeepInterpolation. *bioRxiv*.2020; 10.1101/2020.10.15.34160210.1101/2020.10.15.341602PMC883381434650233

# Response

**Published:** 2021-08-16

**Authors:** Miranda Robbins

**Affiliations:** MRC Laboratory of Molecular Biology, UK

In their review “Calcium imaging analysis – how far have we come?”, Robbins et al. present a concise overview of the basic steps involved in transforming raw microscopy images from calcium imaging experiments to quantifiable data. This is an extremely important topic with broad relevance to many fields. As such, the review should draw considerable interest. The review is well written. It is logically organized, with the analysis pipeline broken down into 4 distinct steps, each of which is discussed separately. The language is easy to follow for a broad audience of varying degrees of expertise. Many of the most popular software implementations are covered at least to some degree. Overall, I think this will be a useful and important review with significant impact.

*We thank Dr. Zeiger for his positive comments about our review and interesting literature references that he has provided.*

 However, I do have some suggestions that I believe could strengthen the manuscript. My main concern is that the target audience for the review is not clear. Calcium imaging is broadly used in many areas of the life sciences, including both in vitro and in vivo preparations, across multiple species, multiple tissues, and widely varying spatial scales (sub-cellular, cellular, bulk tissue signals).

 Given that much of the review is devoted to denoising and motion correction (problems which are relatively minor in in vitro preparations with no organismal movement and relatively high signal-to-noise), and that quantification focuses on spike detection, the review feels most well suited to in vivo two-photon imaging applications in the brain. If this was the intention, it would benefit the review to make this more explicit and provide more discussion tailored to this technique, particularly in the quantification section. If this assumption is not correct and the review is meant to be targeted at a more general audience, I would suggest the introduction be expanded to include at least a brief discussion of the types of calcium indicators available (ratiometric, FRET, fluo, GCaMP, etc) and imaging techniques in use (widefield fluorescence, confocal, 2P, miniscopes, etc). This is important for a beginner/generalist audience as the indicator and imaging technique used will strongly influence the subsequent processing pipeline needs.

*We were indeed targeting the review at a more general reader. We have therefore expanded the regions of the introduction where we mention calcium indicators and imaging techniques as well as including a sentence to discuss differences between in vivo and in vitro samples as follows:*

**“The combination of Ca 2+ indicator and imaging modality used will reflect the properties of the sample and the scientific question, as well as the methodologies available to the researcher. For example, in vitro imaging, or in vivo invertebrate imaging, may use exogenous or GECIs, imaged using LSM, epifluorescence, 2PM or other fluorescence microscopes depending on the temporal-resolution, timescale of imaging, and thickness of the sample being taken into consideration. Other specialist options for in vivo imaging of GECIs are available for imaging in awake and behaving animals including miniaturized forms of 1- or 2- photon fluorescence microscopes (miniscopes) for single-cell in vivo recordings (Chen et al., 2013; Cai et al., 2016; Silva, 2017).**

**As calcium imaging is used across a broad range of samples, from sub-cellular, cellular, networks, bulk tissue dynamics to whole organisms and behaving animals, aspects of this pipeline can vary substantially with no ‘one size fits all’ approach.”**

 In addition to this primary concern I have a few other suggestions that would improve the scope of the review:

- The quantification section should be significantly expanded. This step is the shortest in description, yet arguably can be fraught with the most pitfalls to which investigators fall prey.

*We have expanded the quantification section:*

**“Signal extraction from single cells can be particularly difficult for *in vivo* recordings due to large background fluxes and high spatial overlaps of cells outside of the focus plane. Semi-automated ROI analysis** **(Barbera et al., 2016; Klaus et al., 2017; Pinto & Dan, 2015)** **, principal component analysis independent components analysis (PCA/ICA)** **(Mukamel, Nimmerjahn, & Schnitzer, 2009)** **, and constrained nonnegative matrix factorization (CNMF)** **(Pnevmatikakis et al., 2016; Zhou et al., 2018)** **approaches are techniques that have been explored with different strengths for detecting background and spatial overlap. An ‘extended’ CNMF method has been developed with an adjusted spatiotemporal background model that outperformed PCA/ICA for the simulated and experimental datasets that were tested** **(Zhou et al., 2018)** **. For a package method, the toolbox MIN1PIPE combines a CNMF (Pnevmatikakis et al., 2016) with additional steps to remove false positives (Lu et al., 2018).”**

  

- CaImAn is included under the “classification” section, but includes modules to do more than just classify, including motion correction and registration across imaging sessions

*We have clarified this by adding the sentence:*

**“** **CaImAn is an open-source package with modules for classification, motion correction, source extraction, and spike deconvolution. The classification method is based on convolutional neural networks( Giovannucci et al., 2019).”**

- Spike detection is mentioned, but it should be made explicit that spike detection is completely dependent on knowledge of ground truth about how specific indicators under specific imaging conditions relate and cannot be readily generalized across labs/indicators/preparations. See this recent preprint (Rupprecht et. al https://www.biorxiv.org/content/10.1101/2020.08.31.272450v2 ^1^)

*Thank you for bringing this very interesting preprint to our attention. We appreciate this point and we have included the addition:*

**“The ability to accurately detect spikes requires knowledge of ground truth, usually from electrophysiological recordings. Calcium imaging can be susceptible to variation between neuron type, calcium indicator and its concentration used, the optical resolution, the sampling rate and the noise level. Therefore, it is fundamental to understand how specific indicators react under the given imaging conditions, which cannot be readily generalized across protocols. To try and improve the accuracy of spike detection, a toolkit using a supervised algorithm of spike inference has been developed using a ‘ground truth database’ from a large number of sets of calcium imaging with corresponding electrophysiological measurements (Rupprecht et al., 2021).”**


** **

- On a related point about spike detection, the authors may want to include information about the relative sensitivity (or in some cases lack thereof) of calcium indicators for spike detection (see Hunag et. al https://elifesciences.org/articles/51675 ^2^).

*We have included a discussion in the conclusion about the sensitivity of spike detection:*

**“Calcium imaging for population activity has also been highlighted as an area that requires further research, particularly when imaging over larger fields of view. Using models specific for neuron types imaged may improve detection of Aps, which are commonly under-represented in population activity measurements (Huang et al., 2021). Recent toolboxes with large datasets containing ground-truths may reduce false negatives during analysis (Rupprecht et al., 2021).”**


**  **

- It would be interesting for the authors to comment on 1P miniscope calcium imaging, as this technique is rapidly disseminating and poses specific challenges for imaging analysis. See Lu et. al ( https://www.cell.com/cell-reports/fulltext/S2211-1247(18)30826-X ^3^)

*We have added discussion of miniscopes to Introduction:*

**“Other specialist options for in vivo imaging of GECIs are available for imaging in awake and behaving animals including miniaturized forms of 1- or 2- photon fluorescence microscopes (miniscopes) for single-cell in vivo recordings (Chen et al., 2013; Cai et al., 2016; Silva, 2017).”**

 to the *Motion correction *section:

**“A MathWorks® MATLAB toolbox, miniscope 1-photon imaging pipeline (MIN1PIPE),  has been developed to include denoising, motion correction and signal extraction (Lu et al., 2018). MIN1PIPE motion correction includes several steps including the Lucas-Kanade and Kanade-Lucas-Tomasi (Lucas and Kanade, 1981; Shi and Tomasi, 1994) trackers, and Log-Demons registration (Vercauteren et al., 2009), and outperforms the Lucas-Kanade, Kanade-Lucas-Tomasi, and NoRMcorre for using sample 2-photon videos (Lu et al., 2018).”**

 to the *Quantification* section:

**“For a package method, the toolbox MIN1PIPE combines a CNMF (Pnevmatikakis et al., 2016) with additional steps to remove false positives (Lu et al., 2018).”**


**  **

- Longitudinal tracking of cells across imaging sessions remains a challenge for the field – I am not aware of many algorithms that are widely available to tackle this problem. It could be included in the future directions.

*We have added to the discussion on future challenges:*

**“Longitudinal tracking of specific cells across imaging sessions also remains a challenge so that individual cells can be identified between multiple imaging sessions. A MathWorks® MATLAB toolkit has been made with reported error rates of  < 5 % (Sheintuch et al., 2017); an alternative approach is also available using CaImAn (Giovannucci et al., 2019) though direct comparisons between these methods is difficult without knowing ground truths.”**


**  **

- The Allen institute recently published a preprint on a new denoising technique which looks promising (See Lecoq et. al https://www.biorxiv.org/content/10.1101/2020.10.15.341602v2 ^4^)

*This is an interesting preprint that does indeed seem promising. It can be seen as an extension of the Noise2void method, which we already touch on. We have added the following sentence to the manuscript:*

**“In another recent method, DeepInterpolation** **(Lecoq et al., 2020)** **, the need for ground truth training data is avoided by treating the denoising task as a nonlinear interpolation problem. This assumes the data have a sequential component, such that spatiotemporally overlapping features can be exploited.”**

# Peer review: Reviewer response for version 1

**DOI:** 10.5256/f1000research.54953.r83580

**Published:** 2021-05-05

**Authors:** Christopher Rowlands, Isabell Whiteley

**Affiliations:** 1 Department of Bioengineering, Imperial College London, London, UK

The authors present a brief review of processing and analysis methods for calcium imaging data in neuroscience. The article breaks down the processing steps typically required to view calcium signals from neurons, including denoising, motion correction, and classification, and then the quantification of the data. It appears that the primary contribution of the article is to summarize the most recent and up-and-coming methods for the processing of calcium activity in neurons.

 The abstract describes the intentions of the paper well, bringing the reader up to date on the last 60 years of calcium analysis tools, however several claims are made which are not followed up in the manuscript. For example, the authors do not highlight their preferred methods as they state they will in the abstract, much less provide justification for these choices. The abstract concludes by stating the authors will describe the future needs in the field, and this is successfully done in the conclusion, albeit briefly and without much justification – we would have liked to see some examples of the kinds of groups who might be making progress in these fields, or papers in different fields which might be applicable to calcium activity monitoring.

 Overall we find the contents of this review to be beneficial to users applying calcium imaging to their research, however some additional information or clarification needs to be included or clarified. The following corrections should be made to the review before formal ‘publication’:

 The description of the purpose and benefits of denoising is well explained at the start of the section. In the following sections of motion correction and classification however, the authors proceed directly to the methods used to perform the task, and while this is comprehensive, they fail to justify why these steps are needed in the calcium processing and analysis pipeline. An ‘introduction’ sentence or paragraph for each section is needed.

 The acronym BAPTA should be defined before it is used.

 In the motion-correction section, more distinction should be made between ‘camera-based’ motion correction, where the whole image is captured simultaneously, and ‘scanning-based’ motion correction. There are distinct differences in capabilities and limitations for each approach (for example, scanning-based imaging can suffer from ‘warping’ which affects camera-based imaging much less, but can also sample regions of interest as opposed to the whole image, speeding up imaging to overcome motion artifacts).

 The authors successfully reference the original papers for the different approaches to denoising, motion correction, and classification, but they should also include the references for when the techniques were first applied and used in calcium image analysis, as this is the focus of the review.

 The standard of presentation is OK, but should really be improved before ‘publication’. The first sentence of the abstract is difficult to parse and is not a complete statement. Furthermore, throughout the manuscript there are several difficult-to-parse sentences and some that are ungrammatical (e.g. “to include ratiometric, fluorescence lifetime, or fluorescence intensity, based reporters, and genetically-encoded options […] alongside dyes”, “rendering features to be less defined.”, “ND-SAFIR is a powerful method for removing Poisson-Gaussian noise, which is based on non-local means denoising […] to first use a variance stabilisation step, followed by spatial and temporal patch-based weighted averages of intensity values”, “over multiple days. which can be well rectified using standard registration methods” and so on). Finally, the structure could be improved (“Motion correction can be split into two main categories” really should be followed by an explanation of what those two categories are, and background subtraction is alluded to in the “Quantification” section but not elaborated upon).

 Figure captions should generally be self-contained and comprehensive – they should explain what the reader is expected to observe/pay attention to, and what conclusions should be drawn. Some more elaboration in the figure caption would therefore be welcome.

 Other more minor adjustments that are recommended (but not required) include:

 It would be nice to see a definition of Poisson-Gaussian noise since this is unlikely to be understood by many readers. Similarly clear definition of a ‘patch’ would be helpful, as would explanation of the difference between local and non-local methods.

 As stated previously, further expansion of the methods used for the analysis of calcium signals, such as calculation of dF/F and spike sorting, that are touched on in the quantification section, would be helpful. This section is very brief but would seem to be the section of most interest to the general reader.

 The authors state the paper will describe the methods used for calcium imaging analysis however the focus of the paper is more on the pre-processing methods rather than the analysis. It would be nice to elaborate further on what kinds of analyses are performed on the data after pre-processing – how these calcium traces can be used to infer useful neurobiological information.

 In summary, this is a brief but well-focussed review on a topic that is of interest to several researchers and thus should be formally published after the necessary revisions have been made.

Is the review written in accessible language?

Partly

Are all factual statements correct and adequately supported by citations?

Yes

Are the conclusions drawn appropriate in the context of the current research literature?

Yes

Is the topic of the review discussed comprehensively in the context of the current literature?

Yes

Reviewer Expertise:

Microscopy, instrument development, automation and data analysis, neurophotonics, biophotonics

We confirm that we have read this submission and believe that we have an appropriate level of expertise to confirm that it is of an acceptable scientific standard, however we have significant reservations, as outlined above.

# Response

**Published:** 2021-08-16

**Authors:** Miranda Robbins

**Affiliations:** MRC Laboratory of Molecular Biology, UK

The authors present a brief review of processing and analysis methods for calcium imaging data in neuroscience. The article breaks down the processing steps typically required to view calcium signals from neurons, including denoising, motion correction, and classification, and then the quantification of the data. It appears that the primary contribution of the article is to summarize the most recent and up-and-coming methods for the processing of calcium activity in neurons.

*We thank the reviewers for highlighting areas of improvement in our review article.*

 The abstract describes the intentions of the paper well, bringing the reader up to date on the last 60 years of calcium analysis tools, however several claims are made which are not followed up in the manuscript. For example, the authors do not highlight their preferred methods as they state they will in the abstract, much less provide justification for these choices.

*We acknowledge this inconsistency between the abstract and the content of the manuscript. On closer inspection, we have decided that we do not want to explicitly emphasise our personal preferences and justify them, but rather objectively discuss the strengths of the respective methods and leave it to the reader – particularly as the “best” methods will often be dependent upon the experimental methods used by individual researchers. We have therefore removed the mentioning of “our preferred [methods]” in the relevant sentence in the abstract from:*

**“We will discuss some of the most popular, alongside our preferred, methods for calcium imaging”**

 The abstract concludes by stating the authors will describe the future needs in the field, and this is successfully done in the conclusion, albeit briefly and without much justification – we would have liked to see some examples of the kinds of groups who might be making progress in these fields, or papers in different fields which might be applicable to calcium activity monitoring.

*We have extended the discussion on future challenges to include:*

**“Longitudinal tracking of specific cells across imaging sessions also remains a challenge so that individual cells can be identified between multiple imaging sessions. A MathWorks® MATLAB toolkit has been made with reported error rates of  < 5 % (Sheintuch et al., 2017); an alternative approach is also available using CaImAn (Giovannucci et al., 2019) though direct comparisons between these methods is difficult without knowing ground truths. Calcium imaging for population activity has also been highlighted as an area that requires further research, particularly when imaging over larger fields of view. Using models specific for neuron types imaged may improve detection of APs, which are commonly under-represented in population activity measurements (Huang et al., 2021). Recent toolboxes with large datasets containing ground-truths may reduce false negatives during analysis (Rupprecht et al., 2021).”**

 Overall we find the contents of this review to be beneficial to users applying calcium imaging to their research, however some additional information or clarification needs to be included or clarified. The following corrections should be made to the review before formal ‘publication’:

 The description of the purpose and benefits of denoising is well explained at the start of the section. In the following sections of motion correction and classification however, the authors proceed directly to the methods used to perform the task, and while this is comprehensive, they fail to justify why these steps are needed in the calcium processing and analysis pipeline. An ‘introduction’ sentence or paragraph for each section is needed.

*We have added introductory sentences as follows:*

**“Motion correction is often required for samples due to drift during imaging, or organism movement.”**

**“Classification is required to ensure that the quantification can be performed over specific regions of interest, such as for subcellular area, specific cells, or tissue regions.”**

 The acronym BAPTA should be defined before it is used.

*We have now included this.*

 In the motion-correction section, more distinction should be made between ‘camera-based’ motion correction, where the whole image is captured simultaneously, and ‘scanning-based’ motion correction. There are distinct differences in capabilities and limitations for each approach (for example, scanning-based imaging can suffer from ‘warping’ which affects camera-based imaging much less, but can also sample regions of interest as opposed to the whole image, speeding up imaging to overcome motion artifacts).

*We agree with this suggestion to more clearly outline the types of motion correction described. Most importantly, we want to distinguish between motion that occurs in the imaging system (such as the camera-based and scanning-based motion effects mentioned here) and the more complex motion effects that are due to specimen movement. We have added the following to the “Motion correction” section:*

**“Motion correction is often required** **to ensure consistent image processing across a time stack. We distinguish between two types of motion: (a) drift occurring in the imaging system itself caused by thermal gradients in the microscope, vibrations and mechanical instability (Kreft *et al.*, 2005); (b) subject motion such as fluctuations in the immersion media or the movement of organisms (Jenkinson *et al*., 2002).”**

 The authors successfully reference the original papers for the different approaches to denoising, motion correction, and classification, but they should also include the references for when the techniques were first applied and used in calcium image analysis, as this is the focus of the review.

*We thank the reviewer for this comment. We can appreciate the point that references describing applications of processing methods specific to calcium imaging should be more plentiful. These references are often difficult to find from the existing reviews on calcium imaging and we have therefore tried to add relevant references to this review where possible:*

 For anisotropic filters we have added the references: (Broser et al., 2004; Kitamura and Häusser, 2011). For wavelet thresholding methods we have included: (Besbeas, Feis and Sapatinas, 2004; Wegner, Both and Fink, 2006). We have added: “In the context of calcium imaging, local methods have been shown to perform well (Malik et al., 2011).”.

 For motion correction using rigid methods we have added (Dubbs, Guevara and Yuste, 2016).

 In ‘Classification’ we have added (Malik et al., 2011) as an example of Maximum Likelihood Classification.

 While the calcium imaging literature does not have a lot of examples of non-trivial image processing applications, we have augmented the section with a new reference that indeed does: “Malik, Wasim Q., et al. "Denoising two-photon calcium imaging data." PloS one 6.6 (2011)”.

 The standard of presentation is OK, but should really be improved before ‘publication’. The first sentence of the abstract is difficult to parse and is not a complete statement. Furthermore, throughout the manuscript there are several difficult-to-parse sentences and some that are ungrammatical (e.g. “to include ratiometric, fluorescence lifetime, or fluorescence intensity, based reporters, and genetically-encoded options […] alongside dyes”, “rendering features to be less defined.”, “ND-SAFIR is a powerful method for removing Poisson-Gaussian noise, which is based on non-local means denoising […] to first use a variance stabilisation step, followed by spatial and temporal patch-based weighted averages of intensity values”, “over multiple days. which can be well rectified using standard registration methods” and so on). Finally, the structure could be improved (“Motion correction can be split into two main categories” really should be followed by an explanation of what those two categories are, and background subtraction is alluded to in the “Quantification” section but not elaborated upon).

*Thank you for highlighting these errors. We have edited the sentences as follows:*

**“Techniques for calcium imaging were first demonstrated in the mid-1970s, whilst tools to analyse these markers of cellular activity are still being developed and improved today”**

**“In the past forty years, the methods available for measuring Ca ^2+^ fluxes in cells have expanded to include ratiometric, fluorescence lifetime, or fluorescence intensity-based dyes, and genetically-encoded calcium indicators (GECIs) ( Miyawaki et al., 1997; Ohkura et al., 2005).”**

**“… causing features to appear less well defined.”**

**“ND-SAFIR is a powerful method for removing Poisson-Gaussian noise. It is based on non-local means denoising using a variance stabilisation step, followed by calculating the spatial and temporal patch-based weighted averages for intensity values.”**

**“…view over multiple days, which can be rectified by using standard registration methods.”**

**We have deleted the sentence “Motion correction can be split into two main categories” which has been replaced with the introductory sentence on motion correction.**

**We have expanded the sentence on background subtraction to say:**

**“Multiple methods can be used, including subtracting the intensity values from a region of the image that does not contain Ca2+ indicator from the intensity values in regions of interest. However, care should be taken using background subtraction with ratiometric indicators (Shkryl, 2020).** **F0 baseline values can be calculated by averaging the values before the onset of stimulation in the same region (Galizia, Menzel and Holldobler, 1999), or by low-pass filtering the signal (Balkenius, Bisch-Knaden and Hansson, 2009)(For review Balkenius, Johansson and Balkenius, 2015). “**

 Figure captions should generally be self-contained and comprehensive – they should explain what the reader is expected to observe/pay attention to, and what conclusions should be drawn. Some more elaboration in the figure caption would therefore be welcome.

*We have adapted the Figure caption as follows:*

**“Figure 1. The steps of a common pipeline for calcium imaging analysis can be subdivided into three areas before quantitative analysis is performed. Denoising is an optional step that can help to improve signal-to-noise and enhance features. Motion correction may be necessary in cases of drift or movement. Classification can select regions of interest for which quantitative analysis is performed.**

 “

 Other more minor adjustments that are recommended (but not required) include:

 It would be nice to see a definition of Poisson-Gaussian noise since this is unlikely to be understood by many readers. Similarly clear definition of a ‘patch’ would be helpful, as would explanation of the difference between local and non-local methods.

*Thank you for these suggestions. We agree that these things were not necessarily clear to the general reader, so we have incorporated various changes to address these points. First of all, definitions of the noise sources in fluorescence microscopy and a reference that deals more thoroughly with the subject:*

**“Live-cell imaging generally requires short exposure times and low excitation power to limit the effects of photo-toxicity and photo-bleaching. This leads to image degradation in the form of noise. In fluorescence microscopy the two prevalent noise sources are Poisson noise and Gaussian noise. Poisson noise is caused by the stochastic and discrete nature of photon emission and tends to be dominant at low light levels, whereas Gaussian noise describes the intrinsic thermal and electronic fluctuation in the image sensor (Luisier et al., 2011). “**

*Secondly, non-local methods are now described as using global information, a distinction to local methods that we believe should now make the difference clear. Lastly, we have included a definition of patch as a block of pixels representing a subregion of an image.*

 As stated previously, further expansion of the methods used for the analysis of calcium signals, such as calculation of dF/F and spike sorting, that are touched on in the quantification section, would be helpful. This section is very brief but would seem to be the section of most interest to the general reader.

 The authors state the paper will describe the methods used for calcium imaging analysis however the focus of the paper is more on the pre-processing methods rather than the analysis. It would be nice to elaborate further on what kinds of analyses are performed on the data after pre-processing – how these calcium traces can be used to infer useful neurobiological information.

*We have included the following in the ‘quantification’ section:*

**“Packages will therefore either provide this data of the baseline fluorescence (F0) and deviations from baseline (ΔF), for further analysis, or provide a direct plot.”**

**“Signal extraction from single cells can be particularly difficult for in vivo recordings due to large background fluxes and high spatial overlaps of cells outside of the focus plane which is further increased in 1-photon compared to 2-photon imaging. Semi-automated ROI analysis** **(Barbera et al., 2016; Klaus et al., 2017; Pinto & Dan, 2015)** **, principal component analysis independent components analysis (PCA/ICA)** **(Mukamel et al., 2009)** **, clustering based approaches (like Suite2P;** **Pachitariu et al., 2016)** **, and constrained nonnegative matrix factorization (CNMF)** **(Pnevmatikakis et al., 2016)** **approaches are techniques that have been explored with different strengths for detecting background and spatial overlap. An ‘extended’ CNMF method (CNMF-E) has been developed with an adjusted spatiotemporal background model that outperformed PCA/ICA for the simulated and experimental datasets that were tested** **(Zhou et al., 2018)** **. For a package method, the toolbox MIN1PIPE combines a CNMF** **(Pnevmatikakis et al., 2016)** **with additional steps to remove false positives** **(Lu et al., 2018)** **.** **CaImAn also builds upon the CNMF algorithm** **(Pnevmatikakis et al., 2016)** **to allow it to be fully automated, and CNMF-E for 1-photon endoscopic data** **(Zhou et al., 2018)** **.”**

**“The ability to accurately detect spikes requires knowledge of ground truth, usually from electrophysiological recordings. Calcium imaging can be susceptible to variation between neuron type, calcium indicator and its concentration used, the optical resolution, the sampling rate and the noise level. Therefore, it is fundamental to understand how specific indicators react under the given imaging conditions, which cannot be readily generalized across protocols. To try and improve the accuracy of spike detection, a toolkit using a supervised algorithm of spike inference has been developed using a ‘ground truth database’ from a large number of sets of calcium imaging with corresponding electrophysiological measurements** **(Rupprecht et al., 2021)** **.** **”**

 In summary, this is a brief but well-focussed review on a topic that is of interest to several researchers and thus should be formally published after the necessary revisions have been made.

*We thank you for your positive comments and helpful additions to our article.*
